# Emerging therapies in rheumatoid arthritis: focus on monoclonal antibodies

**DOI:** 10.12688/f1000research.18688.1

**Published:** 2019-08-30

**Authors:** Ladislav Senolt

**Affiliations:** 1 Department of Rheumatology, First Faculty of Medicine, Charles University, Institute of Rheumatology, Prague, Czech Republic, 128 50, Czech Republic

**Keywords:** rheumatoid arthritis, biological therapies, monoclonal antibodies, therapeutic strategies

## Abstract

Advances in the treatment of rheumatoid arthritis (RA) are attributed to several aspects such as new classification criteria enabling early diagnosis and intensive treatment with the application of treat-to-target principles as well as better understanding of the pathogenesis of RA contributing to the development of targeted therapies. However, reaching remission is still not achieved in most patients with RA, which is one of the driving forces behind the continuous development of novel therapies and the optimization of therapeutic strategies. This review will outline several new therapeutic antibodies modulating anti-inflammatory cytokines interleukin (IL)-2 and IL-10 and pro-inflammatory mediators granulocyte-macrophage colony-stimulating factor, fractalkine, and IL-6 that are in various stages of clinical development as well as the progress in manufacturing biotechnologies contributing to the next generation of antibodies and their potential to expand the therapeutic armamentarium for RA. In addition, the fate of unsuccessful therapies including agents targeting IL-15, the IL-20 family, IL-21, chemokine CXCL10, B-cell activating factor (BAFF), and regulatory T (Treg) cells or a novel concept targeting synovial fibroblasts via cadherin-11 will be discussed.

## Introduction

In recent years, dramatic advances have emerged across medical disciplines in understanding the pathogenesis of immune-mediated inflammatory diseases, which are accompanied by a significant shift in treatment options ^1^. One of the most significant advances has been shown in the treatment of rheumatoid arthritis (RA), in which the discovery of glucocorticoid activity was among the first therapeutic milestones in the 1950s ^2^, followed by widespread use of methotrexate since the turn of the 1980s and 1990s ^3^. Later on, success in the treatment of RA can be attributed to the introduction of new classification criteria allowing earlier diagnosis and treatment and application of the treat-to-target principles with the aim to induce remission or at least low disease activity ^4– 6^. The biggest breakthrough in the treatment of RA, however, comes with biologic therapy, which has gradually begun to spread since the beginning of the new millennium with the introduction of tumor necrosis factor (TNF) inhibitors into clinical practice ^7^.

Targeted disease-modifying antirheumatic drugs (DMARDs), consisting of biologic DMARDs (bDMARDs) and targeted synthetic DMARDs (tsDMARDs), antagonize soluble cytokines and their receptors and directly affect immune cell activity or intracellular signaling pathways ^8– 10^. The effect of targeted therapies is relatively rapid and often accompanied by a significant suppression of inflammation. In most cases, it can substantially improve quality of life and stop or significantly slow the progression of functional and structural impairment ^11^. Currently, out of the biological therapies, five original TNF inhibitors and already even more biosimilar TNF inhibitors, two interleukin-6 receptor (IL-6R) inhibitors, and one IL-1 receptor inhibitor as well as B-lymphocyte (CD20) and co-stimulatory signal (CD28-B7 interaction) for T-cell activation inhibitors are available for the treatment of RA ( Table 1). In addition to bDMARDs, two tsDMARDs, including Janus kinase inhibitors (JAKi) tofacitinib (pan-JAKi) and baricitinib (JAK1/2i) ( Table 1), have expanded the treatment armamentarium ^12^.

**Table 1.** Currently approved targeted therapies for rheumatoid arthritis

Original biologic DMARDs		Target	Structure	
Infliximab (3–5 mg i.v. every 8 weeks)		TNF	Chimeric mAb	
Adalimumab (40 mg s.c. every other week)		TNF	Human mAb	
Etanercept (50 mg s.c. every week)		TNF	Fc fusion protein	
Golimumab (50 mg s.c. once a month)		TNF	Human mAb	
Certolizumab pegol (200 mg s.c. every other week)		TNF	Humanized PEGylated Fab fragment	
Rituximab (1,000 mg i.v. every 6 months)		CD20 (B-cells)	Chimeric mAb	
Abatacept (125 mg s.c. every week)		CD80/86 (costimulation)	Fc fusion protein	
Tocilizumab (162 mg s.c. every week)		IL-6R	Humanized mAb	
Sarilumab (150–200 mg s.c. every other week)		IL-6R	Human mAb	
**Targeted synthetic DMARDs**		**Target**	**Structure**	
Tofacitinib (10 mg daily)		JAK1, JAK2, JAK3	Small molecule	
Baricitinib (2–4 mg daily)		JAK1, JAK2	Small molecule	
**Biosimilar DMARDs**	**Target**	**Reference product**	**Approved by EMA/FDA**	**Manufacturer**
Remsima/Inflectra (CT-P13)	TNF	Infliximab	2013/2016	Celltrion
Flixabi/Renflexis (SB2)	TNF	Infliximab	2016/2017	Samsung Bioepis/Biogen
Zessly/Ifixi (GP1111/PF-06438179)	TNF	Infliximab	2018/2017	Sandoz (EU)/Pfizer (US)
Amgevita/Amjevita (ABP 501)	TNF	Adalimumab	2017/2016	Amgen
Cyltezo (BI 695501)	TNF	Adalimumab	2017/2017	Boehringer Ingelheim
Imraldi (SB5)	TNF	Adalimumab	2017/ *	Samsung Bioepis/Biogen
Hyrimoz (GP2017)	TNF	Adalimumab	2018/2018	Sandoz
Hulio (MYL-1401A)	TNF	Adalimumab	2018/2018	Mylan
Benepali (SB4)	TNF	Etanercept	2016/ *	Samsung Bioepis/Biogen
Erelzi (GP2015)	TNF	Etanercept	2017/2016	Sandoz
Truxima (CT P10)	CD20	Rituximab	2017/2018	Celltrion/Hospira (Pfizer)
Rixathon	CD20	Rituximab	2017	Sandoz (EU)

CD, cluster of differentiation; DMARDs, disease-modifying antirheumatic drugs; EMA, European Medicines Agency; FDA, US Food and Drug Administration; IL, interleukin; IL-6R, interleukin-6 receptor; i.v., intravenously; JAK, Janus kinase; mAb, monoclonal antibody; s.c., subcutaneously; TNF, tumor necrosis factor *accepted for review by FDA, assessed 19 March 2019.

Although the fate of patients with RA has improved significantly and a milder course of disease can be observed than in the past ^13^, persistent remission, let alone drug-free remission or complete cure, is still elusive ^14^. This review will outline several new directions for the potential new therapeutic antibodies and treatment strategies for RA, new biological drugs against new targets, and biological drugs against already-known targets that are currently in early or late stages of clinical development.

## Cytokine and cell-targeted therapies

**Figure 1.**
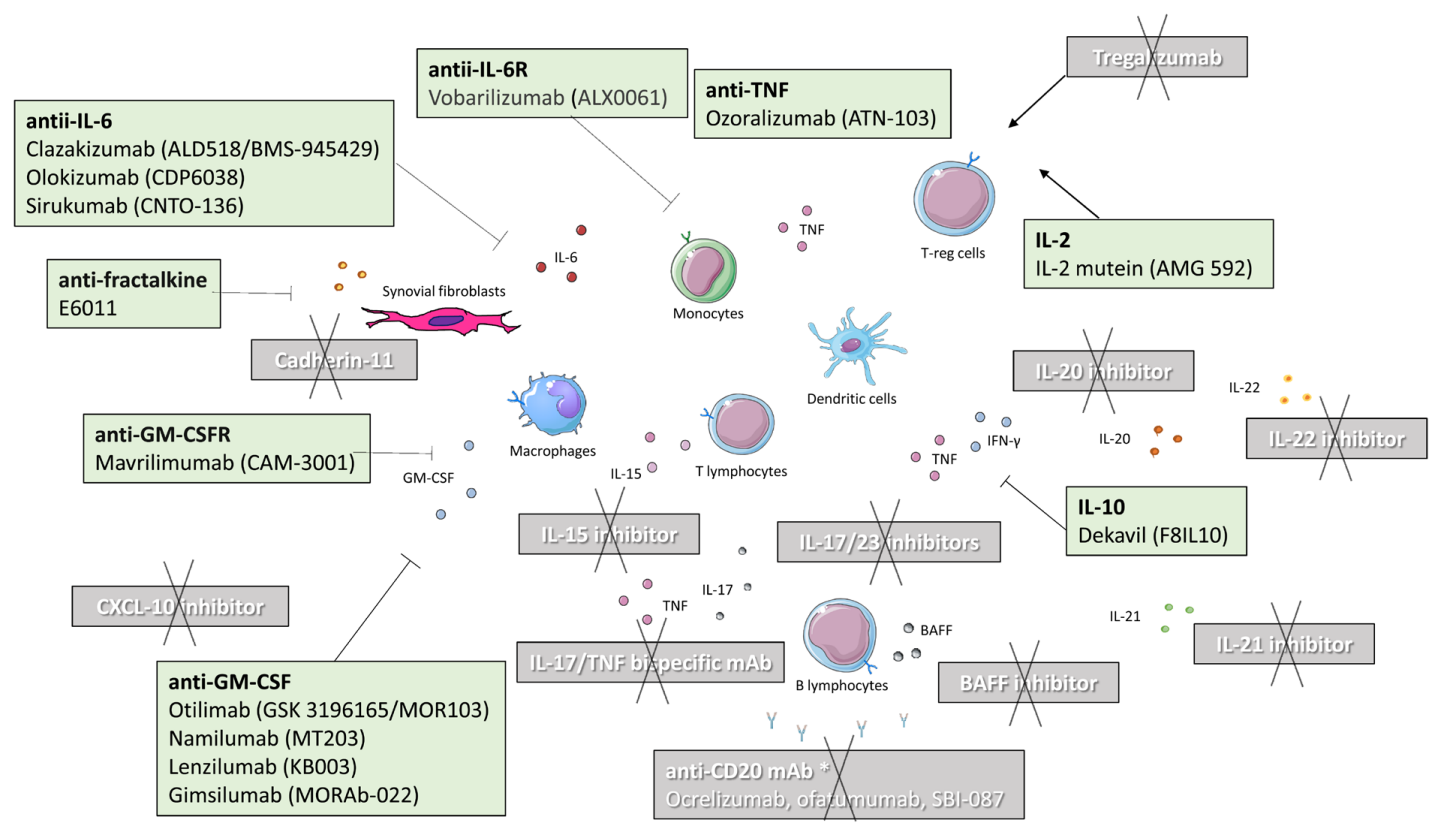
Potential biological therapies for the management of rheumatoid arthritis that are currently in various stages of clinical development. Black text shows therapies that were effective in the treatment of rheumatoid arthritis, although some of the drugs are not further evaluated in rheumatoid arthritis owing to company prioritization. White text shows therapies that failed to prove efficacy or whose clinical trials were terminated owing to safety concerns. *However, anti-CD20 monoclonal antibody such as rituximab is effective and licensed for the treatment of rheumatoid arthritis. BAFF, B-cell activating factor; CXCL, C-X-C motif ligand; GM-CSF, granulocyte-macrophage colony-stimulating factor; GM-CSFR, granulocyte-macrophage colony-stimulating factor receptor; IL, interleukin; mAb, monoclonal antibody; TNF, tumor necrosis factor; Treg, T regulatory

The development of biological therapies for RA has been facilitated by two main assumptions. The first prerequisite is an increasing understanding of the pathophysiological processes in which systemic immune mediators, immune cells, and signaling pathways play a role and have become targets for specific immune interventions ^15^. The second important assumption is the dynamic development of the pharmaceutical industry and advances in genetic engineering and biotechnology ^16^. Potential therapeutic antibodies that are in various stages of clinical development are summarized in Figure 1.

## New targets in later phases of development

### Granulocyte-macrophage colony-stimulating factor

Granulocyte-macrophage colony-stimulating factor (GM-CSF) was first described in the 1970s and is considered a pro-inflammatory cytokine, which is upregulated upon stimulation with multiple mediators such as lipopolysaccharide (LPS) or TNF and is produced by a variety of immune as well as resident tissue cells ^17^. As the expression of GM-CSF is increased in the synovial tissue and synovial fluid of patients with RA, it can stimulate the synthesis of adhesion molecules and pro-inflammatory cytokines, and it can also activate synovial fibroblasts and polarize macrophages into an inflammatory M1 phenotype, thus promoting rheumatoid synovial inflammation ^18^. At present, several monoclonal antibodies targeting the GM-CSF pathway in patients with RA are being studied ^19^.

Mavrilimumab (CAM-3001), a monoclonal antibody against GM-CSF receptor alpha being developed by MedImmune, has been extensively evaluated in the EARTH development program ^20, 21^. Mavrilimumab was administered in a phase II dose-ranging study (10–100 mg) every other week for a total of 12 weeks to patients with active RA on background methotrexate ^20^. The primary endpoint was met: the greater proportion of patients receiving mavrilimumab compared with placebo achieved a ≥1.2 decrease in disease activity score (DAS28-CRP) from baseline at week 12 (55.7% versus 34.7%; *p*=0.003). The 100 mg dose demonstrated a significant effect versus placebo on DAS28-CRP <2.6 (23.1% versus 6.7%, *p*=0.016). In a phase IIb study in RA patients with inadequate response to at least one conventional synthetic DMARD (csDMARD), significantly more mavrilimumab-treated (150, 100, and 30 mg) patients achieved American College of Rheumatology definition of improvement (ACR20) compared with placebo at week 24 (73.4%, 61.2%, 50.6% versus 24.7%, respectively [ *p*<0.001]) and clinically meaningful response was observed as early as one week after treatment initiation ^21^. Another phase IIb study evaluated the efficacy and safety of mavrilimumab and golimumab (anti-TNF) in patients with RA who have had an inadequate response to csDMARDs and anti-TNF agents ^22^. Mavrilimumab demonstrated numerically lower response rates compared with golimumab in patients with inadequate response to csDMARDs and similar efficacy to that of golimumab in the anti-TNF non-responders. However, the study was not powered to provide a direct comparison between mavrilimumab and golimumab, and it is probable that a suboptimal dosage of mavrilimumab was used (100 mg every other week) ^22^. Pulmonary safety assessments were performed because the inhibition of GM-CSF signaling has the potential to affect the clearance of surfactant by alveolar macrophages and thus be responsible for pulmonary alveolar proteinosis. Although there was a potential risk of this rare disease, including neutropenia, infections, and other lung toxicities associated with the blockade of GM-CSF, none of these studies nor a long-term extension study up to 3.3 years revealed such concerns ^23^. Mavrilimumab demonstrated sustained efficacy and an acceptable safety profile; however, due to future clinical development plans, the study was terminated (ClinicalTrials.gov Identifier: NCT01712399).

Otilimab (GSK 3196165 or MOR103) is a fully human high-affinity IgG monoclonal antibody against GM-CSF being developed by GlaxoSmithKline. In a phase IIb dose-ranging study (BAROQUE), patients with active RA were randomized to placebo or otilimab (22.5, 45, 90, 135, or 180 mg) administered weekly for five injections, and then every other week until week 50 ^24^. Dose-related treatment effect with the onset of clinical response as early as week one was observed for all doses. However, the primary endpoint (DAS28 remission at week 24) was not achieved; it was only numerically higher in patients at the highest dose of otilimab versus placebo (16% versus 3%; *p*=0.134). In addition, exposure to otilimab was lower than predicted owing to increased apparent clearance. In July 2019, a phase III clinical program (“ContRAst”) was announced, in which otilimab (90 and 150 mg weekly doses) will be compared with two treatments with different modes of action: JAKi (tofacitinib) and anti-IL-6R (sarilumab) (https://www.gsk.com).

Namilumab (MT203), lenzilumab (KB003), and gimsilumab (MORAb-022) being developed by Takeda, KaloBios Pharmaceuticals, and Morphotek, respectively, are fully human IgG1 monoclonal antibodies against GM-CSF. While initial studies, at least with namilumab ^25^, demonstrated good safety and promising efficacy data, no further results from phase II studies have been reported so far ^19^. In addition, based on negative results from a psoriasis study ^26^ and results from a proof-of-concept study in RA (ClinicalTrials.gov Identifier: NCT02393378), further studies on namilumab in psoriasis and RA were terminated.

Interestingly, GM-CSF-targeting therapies are also being studied in other rheumatic diseases. For instance, otilimab was studied in patients with hand osteoarthritis and showed numerical reduction in pain and functional impairment, while little difference was observed with placebo on tender and swollen joints, including magnetic resonance imaging (MRI) endpoints ^27^. In patients with axial spondyloarthritis, a phase IIa proof-of-concept study evaluating the safety and efficacy of namilumab has been recruiting subjects since August 2018 (ClinicalTrials.gov Identifier: NCT03622658).

## Old targets with the focus on interleukin-6

In mid-2017, sarilumab (trade name Kevzara), a human monoclonal antibody against IL-6R, was approved by the US Food and Drug Administration (FDA) and European Medicines Agency (EMA) for the treatment of RA. Sarilumab showed significantly greater affinity to recombinant human IL-6R compared with tocilizumab and longer half-life with a similar safety profile ^28, 29^. Further biological drugs targeting IL-6 pathways are in development.

Clazakizumab (formerly ALD518 and BMS-945429) is a humanized monoclonal antibody against IL-6. In a phase IIb study ^30^, the primary endpoint was met; ACR20 response rates at week 12 were greater for clazakizumab (25, 100, and 200 mg) once monthly in combination with methotrexate (MTX) or clazakizumab monotherapy (100 and 200 mg) versus MTX (76.3%, 73.3% and 60.0% or 55.0% and 61.0% versus 39.3%; *p*<0.05). Also, remission rates as assessed by DAS28-CRP for clazakizumab plus MTX were greater than for MTX plus original adalimumab (40.0–49.2% versus 23.7%). The treatment was well tolerated with no unanticipated safety signals, consistent with the known pharmacologic effects of IL-6 blockade. Bristol-Myers Squibb has decided, based on portfolio prioritization, to return worldwide rights to clazakizumab to Alder BioPharmaceuticals. However, no further trials in RA are ongoing. The drug is currently in the early phases of development for the treatment of kidney transplant recipients with late antibody-mediated rejection (ClinicalTrials.gov Identifier: NCT03380377 and NCT03380962).

Olokizumab (CDP6038), a humanized monoclonal antibody against IL-6, was formerly developed by UCB Pharma and out-licensed to R-Pharm in 2013. In a phase IIb study, the primary endpoint was met; patients with active RA who had previously failed TNF inhibitor therapy demonstrated a significant decrease in DAS28-CRP at week 12 for all doses (60 mg, 120 mg, and 240 mg) administrated every four or every two weeks with results comparable to tocilizumab ^31^. Safety data were consistent with the use of IL-6-targeted therapy. Although no further results have been reported, phase III studies (CREDO) are either completed (ClinicalTrials.gov Identifier: NCT02760368, NCT02760433) or ongoing (ClinicalTrials.gov Identifier: NCT02760407, NCT03120949).

Sirukumab (CNTO-136; proposed trade name Plivensia), a human monoclonal antibody against IL-6, was developed by Centocor, GlaxoSmithKline, and Janssen Biotech. Sirukumab was investigated at doses of 100 mg and 50 mg every two and four weeks, respectively, in five phase III trials (SIRROUND) in a broad spectrum of patients with RA ^32^. Sirukumab showed significant improvements in disease activity, physical function, and quality of life along with inhibition of radiographic progression ^33, 34^. In a head-to-head study with adalimumab, sirukumab monotherapy showed greater improvements in disease activity (DAS28) but similar ACR50 response rates at week 24 ^35^. Although the long-term safety of sirukumab seems to be similar to that of other IL-6-inhibiting agents, drug regulatory authorities suggested re-evaluating the safety profile of sirukumab for increased mortality rates in post open-label studies ^32^. Interestingly, the drug is currently under development for the treatment of major depressive disorder (ClinicalTrials.gov Identifier: NCT02473289).

## New targets in early phases of development

### Interleukin-2

IL-2, first cloned in the early 1980s, is predominantly produced by CD4 ^+^ T-cells and activated dendritic cells and has pleiotropic functions (for review, see 36). In high doses, IL-2 has been found to promote effector T cells, whereas low-dose IL-2 (ld-IL2) activates regulatory T cells (Tregs) and thus can have a broad therapeutic potential in many autoimmune and inflammatory diseases. Indeed, the results of a first prospective, phase I–IIa clinical trial recently demonstrated that ld-IL2 (1,000,000 IU/day) given for five days and then once every other week for six months selectively activated and expanded Tregs without activating effector T cells. Furthermore, the authors reported signals of efficacy without safety issues in 46 patients across 11 autoimmune and inflammatory chronic diseases, including RA, ankylosing spondylitis, systemic lupus erythematosus (SLE), and psoriasis ^37^. In addition, *in vitro* and first-in-human data on a fusion protein of IL-2 mutein and human Fc (AMG 592) demonstrated dose-dependent, selective expansion of Tregs with no increase of major pro-inflammatory cytokines such as IL-6, TNF, or interferon-γ (IFN-γ) in healthy volunteers ^38^. Based on these data, another phase Ib/IIa study evaluating the safety and efficacy of AMG 592 has been underway in patients with RA since May 2018 (ClinicalTrials.gov Identifier: NCT03410056) but also in patients with SLE (ClinicalTrials.gov Identifier: NCT03451422).

### Interleukin-10

IL-10 is produced by virtually all leukocytes and inhibits the production of pro-inflammatory cytokines, e.g. TNF and IFN-γ, and abrogates antigen presentation and cell proliferation (for review, see 39). Despite the fact that it belongs to the most potent anti-inflammatory cytokine, limited efficacy with subcutaneously administered recombinant IL-10 was observed in a phase I trial in patients with RA in the past ^40^.

Several reasons for this discrepancy can be speculated, e.g. complex mechanism of pathophysiological action of IL-10, including potential pro-inflammatory activity ^41^, or short half-life of IL-10 hampering effective delivery of recombinant IL-10 to the sites of inflammation. To overcome these obstacles, Dekavil (F8IL10), a fully human anti-inflammatory immunocytokine composed of the vascular-targeting anti-fibronectin domain fused to IL-10, is under investigation in patients with RA ^42^. In a phase II clinical trial, Dekavil (30–600 mg/kg) is administered once a week for eight consecutive weeks by subcutaneous injection in combination with MTX to RA patients who have previously failed at least one TNF inhibitor. Preliminary data have demonstrated some signs of efficacy, with 46% demonstrating ACR20 clinical response after eight weeks of drug administration. Dekavil was well tolerated; however, mild injection site reaction occurred in 60% of the patients ^43^.

### Fractalkine

Fractalkine (FKN) is known as a CX3C chemokine that promotes cell adhesion and chemotaxis, but also angiogenesis and osteoclastogenesis, and increases the production of inflammatory mediators, thus playing a significant role in the pathogenesis of RA (reviewed in 44). Recently, first data from a phase II, multicenter, randomized, double-blind, placebo-controlled study with anti-FKN monoclonal antibody (E6011) in patients with active RA were released ^45^. This novel approach targeting FKN demonstrated reliable safety and promising efficacy with a dose-dependent clinical response, particularly in patients who showed higher baseline CD16 ^+^ monocytes (ACR20 at week 24: 30% for placebo, 46.7% for 100 mg, 57.7% for 200 mg, and 69.6% for 400/200 mg).

## Unsuccessful biological therapies in rheumatoid arthritis

Although several pro-inflammatory cytokines play a significant role in the pathogenesis of RA and their inhibition contributed to a significant reduction in synovial inflammation and joint damage in an experimental model of arthritis and proved to be effective in early phases of development in humans, further studies failed to confirm significant efficacy ^46^ ( Table 2). For instance, IL-1 inhibitors are approved but only modestly effective in RA while highly effective in several autoinflammatory diseases ^47^. An early phase study with IL-15 inhibitor therapy seemed to be efficient ^48^, but a phase II clinical trial of a fully human monoclonal antibody against IL-15 failed to confirm significant efficacy (ClinicalTrials.gov Identifier: NCT00433875). Although targeting the IL-23/17 axis is effective in spondyloarthritis ^49^, strategies to block the IL-17 pathway, IL-12/23 p40, or IL-23 did not prove to be effective in patients with established RA, and the clinical research programs in RA were discontinued ^50^. Similarly, the IL-20 family of cytokines such as IL-20 and IL-22 play a significant role in the process of immune cell activation and bone destruction, and although an early phase trial with the IL-20 inhibitor fletikumab was well tolerated and effective, particularly in patients with seropositive RA, further studies with fletikumab and also IL-22 inhibitor fezakinumab were completed several years ago with negative or no final results released ^51, 52^. Although IL-21 plays an important role in the activation of the immune system, an early phase first-in-man trial with an IL-21 inhibitor (NNC0114-0000-0005) in patients with RA and healthy subjects was finished in 2012 and no further results have been released (ClinicalTrials.gov Identifier: NCT01208506). The first study demonstrating good safety and clinical efficacy of a chemokine inhibitor in patients with RA evaluated eldelumab (MDX-1100), a fully human anti-CXCL10 (anti-IP-10) monoclonal antibody ^53^; however, no further data were released.

**Table 2.** Selected unsuccessful biological therapies in rheumatoid arthritis

Drug	Target	Reason for failure	Citation
HuMax-IL15 (anti-IL-15 mAb)	IL-15	Lack of efficacy	*
Brodalumab (anti-IL-17R mAb)	IL-17	Lack of efficacy	50
Secukinumab (anti-IL-17A mAb)	IL-17	Lack of efficacy	50
Ixekizumab (anti-IL-17A mAb)	IL-17	Lack of efficacy	50
Bimekizumab (dual anti-IL-17A/F mAb)	IL-17	Lack of efficacy	50
Ustekinumab (anti-IL-12/23p40mAb)	IL-12/IL-23	Lack of efficacy	50
Guselkumab (anti-IL-23 mAb)	IL-23	Lack of efficacy	50
Remtolumab (dual anti-TNF/IL-17A)	TNF/IL-17	Not higher efficacy than adalimumab	67
NNC0114 (anti-IL-21 mAb)	IL-21	No final results released	+
Fletikumab (anti-IL-20 mAb)	IL-20	Lack of efficacy	51
Fezakinumab (anti-IL-22 mAb)	IL-22	Negative or no final results released	51
Eldelumab (anti-CXCL10 mAb)	CXCL10	Effective/safe, but no final results released	53
Mavrilimumab (anti-GM-CSFRα)	GM-CSFRα	Effective/safe, terminated (sponsor decision)	ɸ
Namilumab (anti-GM-CSF)	GM-CSF	Lack of efficacy	♯
Tabalumab (anti-BAFF)	BAFF	Lack of efficacy	56
Belimumab (anti-BAFF)	BAFF	Terminated (sponsor decision)	±
Ocrelizumab (anti-CD20 mAb)	B-cells	Risk/benefit consideration	54
Ofatumumab (anti-CD20 mAb)	B-cells	Risk/benefit consideration	54
SBI-087 (CD20-targeted SMIP)	B-cells	Further development terminated	55
Alemtuzumab (anti-CD52 mAb)	T-cells	Safety	57
Keliximab (anti-CD4 mAb)	T-cells	Safety	57
Clenoliximab (anti-CD4 mAb)	T-cells	Safety	57
Tregalizumab (anti-CD4 mAb)	Treg cells	Lack of efficacy	58
RG6125 (anti-cadherin-11 mAb)	Cadherin-11	Lack of efficacy	59

BAFF, B-cell activating factor; CXCL, chemokine C-X-C motif ligand; GM-CSFRα, granulocyte-macrophage colony-stimulating factor receptor α; IL, interleukin; mAb, monoclonal antibody; SMIP, Small Modular ImmunoPharmaceutical; TNF, tumor necrosis factor; Treg, T regulatory * ClinicalTrials.gov Identifier: NCT00433875 + ClinicalTrials.gov Identifier: NCT01208506 ɸ ClinicalTrials.gov Identifier: NCT01712399 ♯ ClinicalTrials.gov Identifier: NCT03622658 ± ClinicalTrials.gov Identifier: NCT00583557

Several strategies to inhibit B-cells were tested; for instance, humanized and human monoclonal antibodies ocrelizumab (trade name Ocrevus) and ofatumumab (trade name Arzerra) proved to be effective in patients with RA. However, after risk/benefit consideration, further studies were discontinued ^54^. In order to reduce the molecule, promote penetration to sites of inflammation, and improve efficacy, single-chain antibody polypeptides known as SMIPs (small modular immunopharmaceuticals) have been developed that are approximately one-half to one-third of the size of the standard monoclonal antibody. A next-generation, humanized SMIP protein therapeutic against the CD20 antigen (SBI-087) was evaluated in patients with RA, but further development was terminated ^55^. In addition, a further strategy to inhibit B-cell differentiation and survival via anti-B-cell activating factor (anti-BAFF) antibody has also failed in the treatment of patients with RA ^56^. Direct T-cell-targeting therapies such as alemtuzumab, keliximab, and clenoliximab did not show satisfactory results and were accompanied by a number of serious adverse reactions associated with severe CD4 ^+^ T-lymphopenia and rash ^57^. Tregalizumab (BT-061) is a humanized agonist antibody that selectively activates Treg lymphocytes and was tested as an innovative treatment for RA; however, it also did not show significant clinical benefit ^58^.

Recently, a novel approach targeting synovial fibroblasts in RA via the inhibition of cadherin-11 was presented ^59^. Although an interesting concept, anti-cadherin-11 (RG6125) given on top of anti-TNF therapy in patients with active RA failed to be more effective than placebo and the study was discontinued ^59^.

## Next-generation antibodies

Conventional monoclonal antibodies (e.g. infliximab or adalimumab), Fc fusion proteins (etanercept and abatacept), and one antibody-derived molecule, a PEGylated anti-TNF Fab fragment (certolizumab pegol), are widely used for the treatment of patients with RA. Further advances in antibody engineering have led to the development of so-called next-generation therapeutic antibodies representing antibody fragments, dual/bispecific targeting variants, and immunocytokines ^60– 62^. The purpose is to develop antibodies that are smaller, more stable, have higher affinity and improved tissue penetration, and have lower immunogenicity and toxicity than conventional antibodies. For instance, nanobodies are single-domain antibodies that were originally developed after the discovery of camelid antibodies consisting of heavy chain variable domain (lacking the Fc-equivalent region) and can be engineered to bind to two (or even more) different antigens or epitopes on the cell surface, so-called bispecific (even multispecific) antibodies ^61, 62^. These antibodies are frequently used to treat cancer. Another therapeutic success was achieved by the development of immunocytokines, a fusion of an antibody and cytokine, which are widely studied as an anticancer therapy ^63^ and should extend cytokine half-life, reduce systemic adverse events, and deliver the cytokine specifically to sites of inflammation as mentioned above (Dekavil or AMG 592).

Belgian biopharmaceutical company Ablynx has developed unique new-generation bispecific therapeutic nanobodies ozoralizumab (ATN-103) and vobarilizumab (ALX0061) for the treatment of RA, the first humanized bispecific nanobodies, which neutralize TNF and IL-6R, respectively, and also bind to human serum albumin to increase the half-life. Ozoralizumab was tested in subcutaneous form at a once-monthly dosing interval in patients with RA and after 12 weeks of treatment, EULAR response was obtained in 97% of patients, an ACR20 response was achieved in 84%, and DAS28 remission was seen in 38% of patients with RA ^64^. A phase II study evaluating long-term safety and tolerability was completed in 2012 (ClinicalTrials.gov identifier: NCT01063803), and no further reports of development have been made so far. An early phase study with vobarilizumab demonstrated promising results as well, with ACR20 response rates of up to 84% and DAS28 remission rates of up to 58% ^65^. In addition, in a phase IIb monotherapy study (head-to-head versus tocilizumab) in RA patients ^66^, a similar proportion of vobarilizumab-treated patients achieved ACR20 response at week 12 compared with tocilizumab (73–81% versus 78%). Although more patients achieved DAS remission on highest vobarilizumab dose (225 mg every other week), when using more stringent CDAI and SDAI remission criteria, a similar efficacy was obtained in the monotherapy with vobarilizumab (5–10%) and tocilizumab groups (9–11%).

Remtolumab (ABT-122), developed by AbbVie, is another bispecific antibody, a full-length IgG with dual-variable domains (DVD-Ig), targeting two important human cytokines: TNF and IL-17A. Based on a TNF-transgenic mouse model of arthritis, dual inhibition of both cytokines proved to be more effective in suppressing inflammation and bone/cartilage destruction than either cytokine inhibitor alone ^67^. However, a phase II trial demonstrated that a strategy of dual inhibition of TNF and IL-17A with remtolumab does not appear to be significantly different from that of TNF inhibition alone with adalimumab in human patients with RA and psoriatic arthritis ^68^. In addition to this finding, recent data proved that another DVD-Ig lutikizumab (ABT-981) targeting IL-1α/β did not improve pain or synovitis in patients with both erosive hand osteoarthritis and knee osteoarthritis ^69, 70^.

## Conclusion

Biologicals have become an essential part of the therapeutic armamentarium for RA, significantly ameliorating disease activity and improving quality of life. Furthermore, the progress in our understanding of the pathogenesis of RA has significantly contributed to the development of novel intracellular targeted therapies such as JAK inhibitors tofacitinib and baricitinib that have already been implemented in routine clinical practice. Nowadays, more selective JAK inhibitors and other targeted synthetic therapies are in various stages of clinical development in RA, which is beyond the scope of this review and is reported elsewhere ^71, 72^. In addition to the development of new therapies, the patent protection of older original biological agents expires and biosimilars have been entering the market within the last few years, thus lowering the price and enabling the treatment of more patients ^73^.

Despite current practice, early and intensive management with the treat-to-target approach, reaching remission is still not achieved in most patients, which is one of the driving forces behind the continuous development of novel therapies and the optimization of therapeutic strategies ^74^. Thus, advances in antibody engineering and further therapeutic targets such as neuro-immune modulation, gene therapy, and epigenetic modification are being evaluated in the treatment of RA ^75– 77^. In addition, the two main treatment strategies can be potentially applied in the future to improve disease outcome, halt further progression, or even cure the disease either 1) by an induction-maintenance regime with biological therapies administered as early as the diagnosis is established ^78^ or 2) even before clinical arthritis becomes apparent ^79^. Currently, several ongoing proof-of-concept trials are testing whether targeting T-cells or B-cells can induce immunological reset and be used to prevent the onset of RA ^80^. An advanced study demonstrated that a single infusion of 1,000 mg rituximab significantly delayed the development of arthritis in patients at risk of developing of RA ^81^. Other treatment approaches for preventive strategies may represent, for instance, the use of tolerogenic dendritic cells ^82^ or targeting the IL-17/23 axis ^83^ in order to block arthritis development in pre-clinical phases of the disease. However, further research is needed to determine which therapeutic agent or strategy would best suit each individual patient.
